# Primary Tumor Resection and Survival Benefit in Patients with Synchronous Metastatic Primary Malignant Bone Neoplasms: A Propensity Score-Matched Analysis of the SEER Database

**DOI:** 10.3390/cancers18142201

**Published:** 2026-07-08

**Authors:** Junjie Bao, Qingyu Shi, Mingbo Liu, Huimin Xu, Yunzhou Wu, Jingya Zeng

**Affiliations:** 1 Department of Orthopedic Surgery, Harbin Medical University Cancer Hospital, Harbin 150081, China; baojunjie@hrbmu.edu.cn (J.B.); 830945@hrbmu.edu.cn (Q.S.); 2024021847@hrbmu.edu.cn (M.L.); 2 Department of Clinical Laboratory, Harbin Medical University Cancer Hospital, Harbin 150081, China; 2019021504@hrbmu.edu.cn; 3 College of Life Science, Northeast Agricultural University, Harbin 150030, China

**Keywords:** primary malignant bone neoplasms, primary tumor resection, propensity score matching, SEER database, survival analysis

## Abstract

**Background**: The survival benefit of primary tumor resection (PTR) in patients with synchronous metastatic primary malignant bone neoplasms (PMBNs) remains controversial. We aimed to evaluate the association between PTR and survival outcomes using a large population-based cohort with rigorous confounding control. **Methods**: Patients diagnosed with synchronous metastatic PMBNs (osteosarcoma, chondrosarcoma, Ewing sarcoma, and chordoma) between 2004 and 2022 were identified from the Surveillance, Epidemiology, and End Results (SEER) database. Patients were stratified by receipt of PTR. Propensity score matching (PSM; 1:1 nearest-neighbor, caliper = 0.03) was applied to balance baseline covariates. Kaplan–Meier analysis with log-rank testing and multivariable Cox proportional hazards regression stratified by matched pairs were used to assess overall survival (OS) and cancer-specific survival (CSS). Subgroup analyses were performed across four histological subtypes. **Results**: A total of 1046 patients were included (resection: *n* = 658, 62.9%; no resection: *n* = 388, 37.1%). After PSM, 488 patients (244 per group) were retained. In the matched cohort, PTR was independently associated with significantly improved Overall survival (OS) (HR = 0.34, 95% CI: 0.21–0.54, *p* < 0.001) and cancer-specific survival (CSS) (HR = 0.35, 95% CI: 0.22–0.55, *p* < 0.001). Subgroup analyses demonstrated significant survival benefit in osteosarcoma and chondrosarcoma (both *p* < 0.001), but not in Ewing sarcoma or chordoma. **Conclusions**: PTR is associated with a significant survival benefit in selected patients with synchronous metastatic PMBNs, particularly those with osteosarcoma and chondrosarcoma. These findings support individualized, multidisciplinary decision-making regarding surgical intervention in this population.

## Simple Summary

Cancer that starts in the bone and has already spread to other parts of the body at the time of diagnosis is difficult to treat. Doctors are unsure whether removing the original bone tumor through surgery helps these patients live longer. In this study, researchers used a large cancer database to compare survival between patients who had their primary bone tumor removed and those who did not. The results show that removing the tumor was linked to longer life, especially in patients with osteosarcoma and chondrosarcoma. These findings may help doctors and families make better treatment decisions when facing advanced bone cancer.

## 1. Introduction

Primary malignant bone neoplasms (PMBNs) are a heterogeneous group of rare but aggressive malignancies that collectively impose a significant global health burden. The four major histological subtypes, osteosarcoma, chondrosarcoma, Ewing sarcoma, and chordoma, differ substantially in their biological behavior, age distribution, and treatment responsiveness [1,2,3]. Osteosarcoma is the most common primary bone malignancy, predominantly affecting children and adolescents, with a peak incidence in the second decade of life [4]. Ewing sarcoma is the second most common bone sarcoma in pediatric and young adult populations, while chondrosarcoma predominantly affects adults over 40 years of age [5]. Chordoma, arising from notochordal remnants, is exceedingly rare and typically presents in the axial skeleton of middle-aged and older adults [6]. Globally, the age-standardized incidence of bone cancer has remained relatively stable over recent decades, with an estimated incidence of approximately 3–5 cases per million person-years [1,2,6]. Despite advances in multimodal therapy, the overall 5-year survival rate for PMBNs remains approximately 60–70% for localized disease but drops precipitously to less than 20–30% in patients presenting with synchronous distant metastasis [3,7,8]. Approximately 15–20% of patients with PMBNs harbor distant metastases at initial diagnosis, with the lungs representing the most common metastatic site [8,9]. This metastatic presentation confers a dramatically worse prognosis, substantially diminishing patients’ quality of life and placing considerable demands on healthcare resources [1,7].

The optimal treatment strategy for patients with synchronous metastatic PMBNs remains a subject of considerable debate. Current management typically involves systemic chemotherapy as the cornerstone of treatment, particularly for osteosarcoma and Ewing sarcoma, where cytotoxic regimens such as MAP (methotrexate, doxorubicin, and cisplatin) and VDC/IE (vincristine, doxorubicin, cyclophosphamide alternating with ifosfamide and etoposide) are standard [10,11]. Radiotherapy plays a central role in Ewing sarcoma, while targeted therapies and immunotherapy remain investigational for most subtypes [12,13]. Surgical resection of the primary tumor has long been the standard of care for localized PMBNs, but its role in the metastatic setting is far less established [14,15]. Some studies have suggested that PTR may reduce tumor burden, eliminate the primary source of metastatic seeding, and improve systemic disease control, thereby translating into survival benefit [16,17]. Conversely, other investigators have argued that local surgery offers limited benefit in the context of systemic disease, particularly when metastatic burden is high or when patients have poor performance status [18,19]. Prior studies addressing this question have been substantially limited by small sample sizes, single-center retrospective designs, marked heterogeneity in patient populations, and critically, significant treatment selection bias, as patients selected for surgery tend to be younger and have better performance status than those managed non-operatively [14,16,19]. The absence of large-scale, population-based evidence with rigorous confounding control has left clinicians without clear guidance for this challenging clinical scenario.

To address these limitations, we leveraged the Surveillance, Epidemiology, and End Results (SEER) database, which captures cancer incidence, treatment, and survival data from approximately 30% of the United States population and provides one of the largest available repositories of real-world oncological data [16,17,20]. To minimize treatment selection bias, the principal methodological challenge in this field, we employed propensity score matching (PSM), a well-validated statistical approach that balances observed baseline covariates between surgical and non-surgical groups, thereby enabling a more valid comparison of outcomes [21,22]. The primary aim of this study was to evaluate whether PTR is associated with improved OS and CSS in patients with synchronous metastatic PMBNs. We further hypothesized that the survival benefit of PTR may vary across histological subtypes and therefore conducted pre-specified subgroup analyses stratified by tumor type. This study represents one of the largest population-based, PSM-controlled, histotype-stratified analyses of PTR in metastatic PMBNs to date and aims to provide actionable evidence to inform multidisciplinary treatment decision-making.

## 2. Materials and Methods

### 2.1. Data Source and Study Population

**Figure 1:**
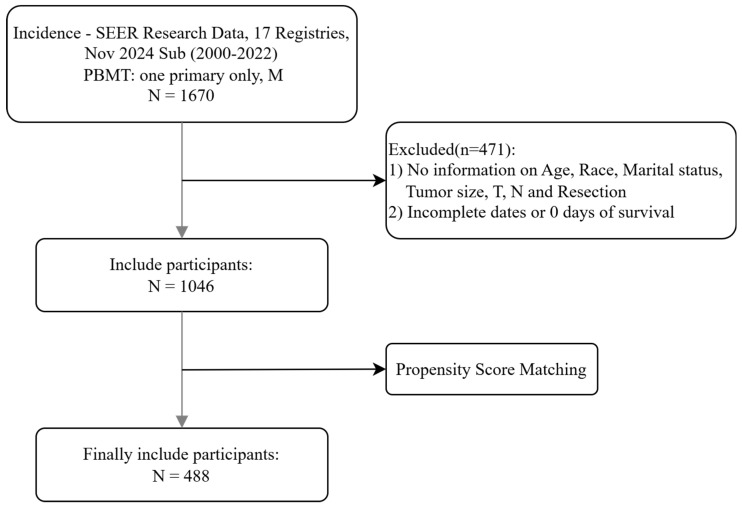
Flowchart depicting the participant selection process from the SEER database. Patients with primary malignant bone neoplasms (osteosarcoma, chondrosarcoma, Ewing sarcoma, and chordoma) diagnosed between 2004 and 2022 were screened. Exclusion criteria included unknown age or race, unknown marital status, multiple primary tumors, absence of distant metastasis, missing tumor size, and incomplete survival data. The final analytic cohort comprised 1046 patients, of whom 488 were retained after 1:1 propensity score matching.

Data were extracted from the SEER database (Incidence—SEER Research Data, 17 Registries, November 2024 Sub, 2004–2022). Patients with a primary diagnosis of osteosarcoma, chondrosarcoma, Ewing sarcoma, or chordoma who presented with synchronous distant metastasis (M1 stage) at initial diagnosis were eligible. Distant metastasis was defined using AJCC M-staging variables appropriate to each diagnostic year (AJCC 6th edition for 2004–2015, AJCC 7th edition for 2010–2015, SEER Combined M for 2016–2017, and EOD 2018 M Recode for 2018 onwards). Inclusion criteria required: known age and race, known marital status at diagnosis, a single primary tumor, presence of distant metastasis, complete tumor size information, and available survival data (survival time > 0 days). Patients were classified as having undergone PTR or not based on SEER surgery codes (Figure 1).

Chordoma was included in the study population because it meets the prespecified inclusion criteria as a primary malignant bone neoplasm in the SEER database. Including chordoma allowed us to present a broader population-based overview of synchronous metastatic primary malignant bone tumors. However, we acknowledge that chordoma differs substantially from osteosarcoma, Ewing sarcoma, and chondrosarcoma in terms of biology, natural history, and treatment strategy. Chordoma is a notochordal tumor with distinct molecular characteristics, typically slow-growing, and primarily managed with surgery and proton/carbon-ion radiotherapy rather than conventional chemotherapy. Given these biological differences and the small number of chordoma cases in our cohort, the chordoma subgroup findings should be considered exploratory and interpreted with caution.

### 2.2. Covariates

The exposure variable was receipt of PTR (yes/no). Covariates included age group (<20, 20–59, ≥60 years), sex, race (White, Black, Other), marital status (married, other), histological type, primary site (axial vs. extremity), tumor grade (I–II, III–IV, unknown), T stage, N stage, tumor size (<5, 5–15, >15 cm), chemotherapy (yes/no), and radiotherapy (yes/no). Neoadjuvant and adjuvant radiotherapy were additionally recorded.

Marital status was included as a covariate because it is an available sociodemographic variable in the SEER database. In retrospective population-based analyses, including available variables that may be associated with both treatment receipt and prognosis can help reduce measurable confounding. We acknowledge that marital status is not a tumor-specific biological factor and cannot directly represent more detailed measures such as socioeconomic status, caregiver support, performance status, or healthcare access; its inclusion is therefore intended as a proxy for social support and healthcare access rather than as a direct clinical determinant.

### 2.3. Propensity Score Matching

To reduce treatment selection bias, PSM was performed using the MatchIt package in R 4.5.0. Propensity scores were calculated using a multivariable logistic regression model that included age category, sex, race, marital status, histologic subtype, tumor location, tumor grade, T stage, N stage, chemotherapy and radiotherapy as covariates. Nearest-neighbor 1:1 matching was applied with a caliper of 0.03 on the logit scale (method = “nearest”, ratio = 1, caliper = 0.03, distance = “logit”). Balance was assessed by comparing standardized mean differences (SMDs) and baseline characteristics before and after matching using chi-squared or Fisher’s exact tests; the SMD distribution is shown in Figure S1.

### 2.4. Survival Analysis

Overall survival (OS) was measured from the date of diagnosis to death from any cause, whereas cancer-specific survival (CSS) was defined as the interval from diagnosis to death caused by the primary malignancy. In both the unmatched and matched cohorts, survival distributions were estimated using Kaplan–Meier methods and compared with log-rank tests. Multivariable Cox proportional hazards regression was performed in the PSM cohort, with stratification by matched pair (subclass) to account for the matched design. A stepwise selection strategy was applied: variables with *p* < 0.1 in univariable analysis were entered into the multivariable model. Results are reported as hazard ratios (HRs) with 95% confidence intervals (CIs).

### 2.5. Subgroup Analysis

Kaplan–Meier survival curves were generated separately for each of the four histological subtypes within the PSM cohort. Multivariable Cox models were fitted within each subtype where sample size permitted. A forest plot was constructed to visualize subtype-specific HRs. Interaction between PTR and histological subtype was tested using the likelihood ratio test comparing models with and without a PTR × histology interaction term.

### 2.6. Statistical Analysis

All analyses were performed using R version 4.5.0. All tests were two-sided; *p* < 0.05 was considered statistically significant.

## 3. Results

### 3.1. Patient Selection and Baseline Characteristics

A total of 1046 patients with synchronous metastatic PMBNs were identified from the SEER database (2004–2022). Of these, 658 (62.9%) underwent PTR and 388 (37.1%) did not. Before PSM, the two groups differed significantly in age distribution, histological type, primary site, tumor grade, N stage, and radiotherapy use (all *p* < 0.05; Table 1). Notably, younger patients (<20 years) were more prevalent in the resection group (55.78% vs. 32.73%), osteosarcoma was more common in the resection group (60.64% vs. 36.86%), and Ewing sarcoma was more prevalent in the non-resection group (42.78% vs. 19.60%). Extremity tumors predominated in the resection group (75.53% vs. 39.69%), while axial tumors were more common in the non-resection group (60.31% vs. 24.47%). After 1:1 PSM, 488 patients (244 per group) were retained. Most baseline characteristics were well balanced between groups (all *p* > 0.05), including age, sex, race, marital status, histological type, primary site, grade, T stage, N stage, tumor size, chemotherapy, and overall radiotherapy (Table 2).

### 3.2. Survival Outcomes Before PSM

**Figure 2:**
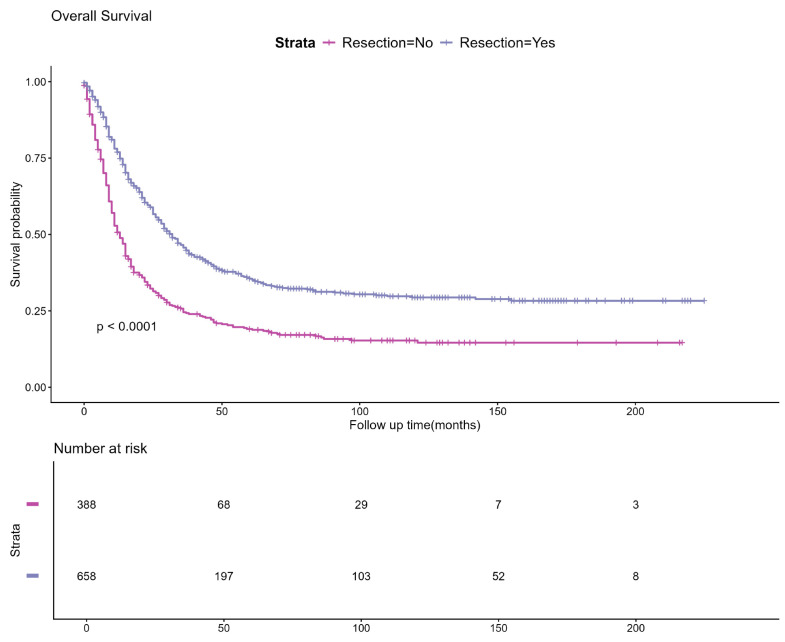
Kaplan–Meier curves for overall survival (OS) stratified by primary tumor resection status in patients with synchronous metastatic primary malignant bone neoplasms before propensity score matching (PSM). The resection group demonstrated significantly superior OS compared with the non-resection group (log-rank *p* < 0.001).

**Figure 3:**
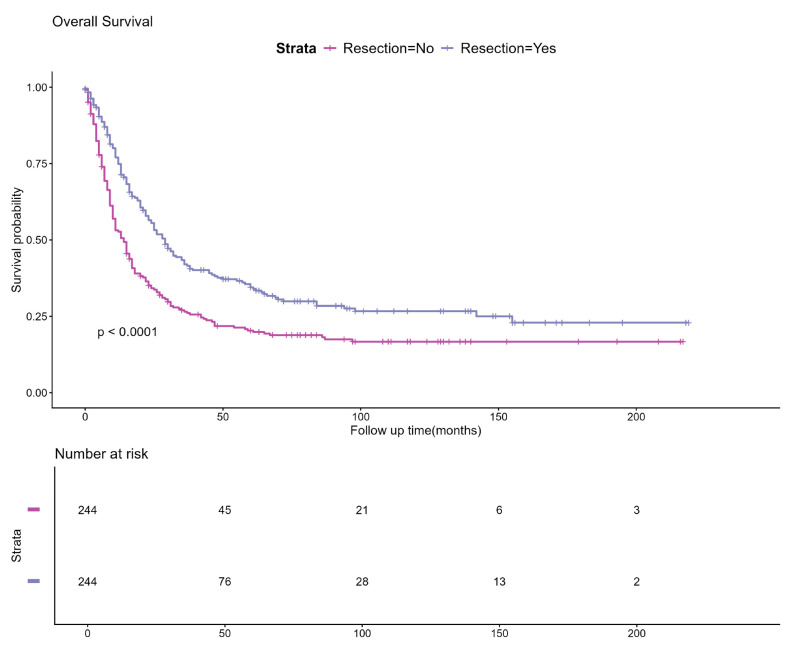
Kaplan–Meier curves for cancer-specific survival (CSS) stratified by primary tumor resection status before propensity score matching (PSM). The resection group demonstrated significantly superior CSS compared with the non-resection group (log-rank *p* < 0.001).

In the unmatched cohort, Kaplan–Meier analysis demonstrated significantly superior OS (Figure 2) and CSS (Figure 3) in the resection group compared with the non-resection group (both log-rank *p* < 0.001).

### 3.3. Survival Outcomes After PSM

**Figure 4:**
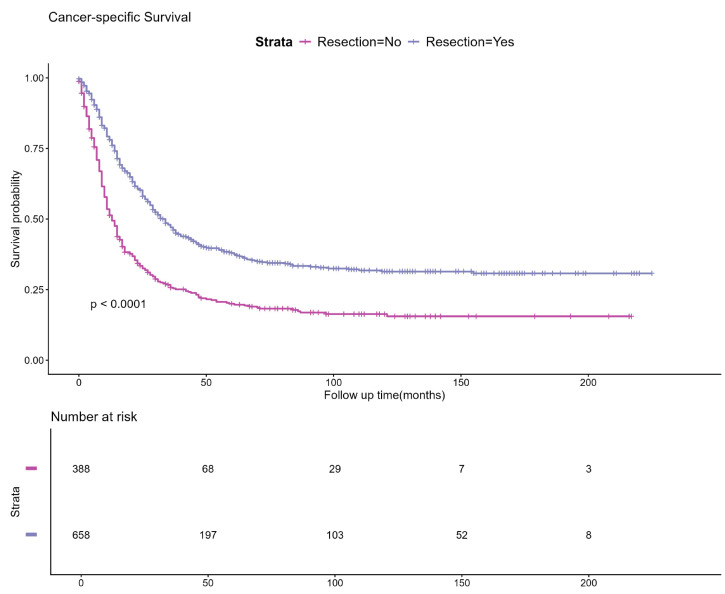
Kaplan–Meier curves for overall survival (OS) stratified by primary tumor resection status after propensity score matching (PSM). In the matched cohort (*n* = 488), the resection group maintained significantly superior OS (log-rank *p* < 0.001).

**Figure 5:**
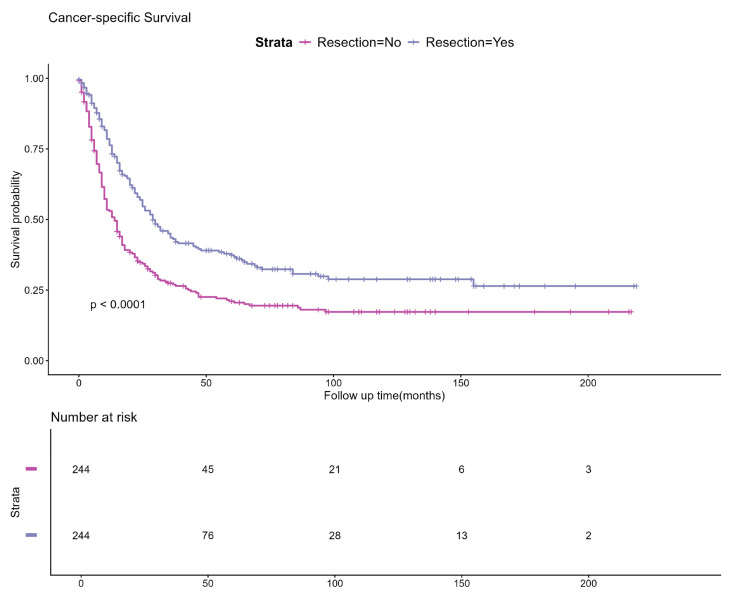
Kaplan–Meier curves for cancer-specific survival (CSS) stratified by primary tumor resection status after propensity score matching (PSM). In the matched cohort (*n* = 488), the resection group maintained significantly superior CSS (log-rank *p* < 0.001).

In the matched cohort (*n* = 488), the resection group continued to demonstrate significantly superior OS (Figure 4) and CSS (Figure 5) compared with the non-resection group (both log-rank *p* < 0.001).

### 3.4. Cox Regression Analyses

In the PSM cohort, multivariable Cox regression for OS (*n* = 488, events = 351) identified PTR as a significant independent protective factor (HR = 0.34, 95% CI: 0.21–0.54, *p* < 0.001). High-grade tumors (Grade III–IV) were associated with significantly increased mortality risk compared with Grade I–II tumors (HR = 2.61, 95% CI: 1.00–6.77, *p* = 0.049; Table 3). For CSS (*n* = 488, events = 338), results were highly consistent: PTR remained a significant independent predictor of improved CSS (HR = 0.35, 95% CI: 0.22–0.55, *p* < 0.001). In addition, patients with chordoma demonstrated significantly lower cancer-specific mortality than those with osteosarcoma. (HR = 0.11, 95% CI: 0.01–0.94, *p* = 0.043; Table 4). Taken together, PTR was the most robust independent prognostic factor across both survival endpoints.

### 3.5. Subgroup Analyses by Histological Subtype

**Figure 6:**
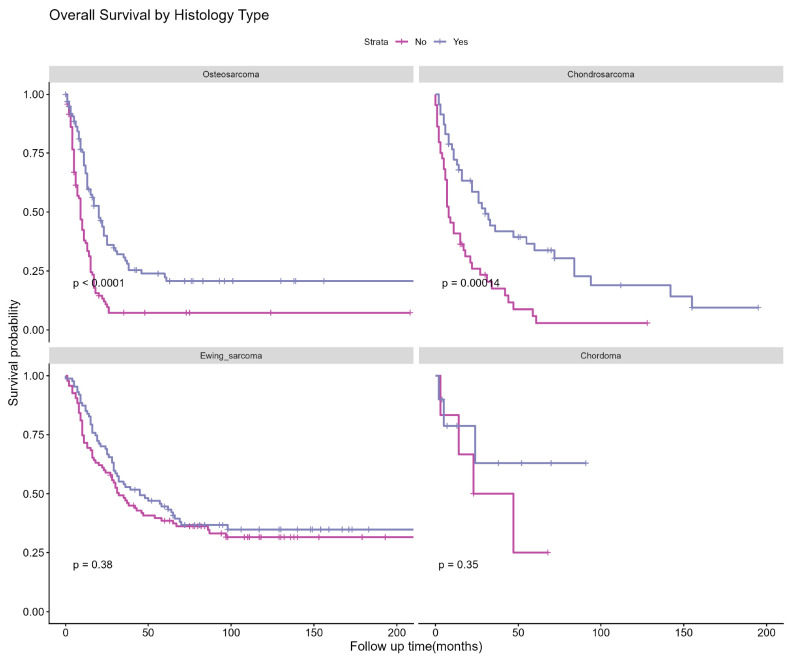
Kaplan–Meier curves for overall survival (OS) stratified by primary tumor resection status across four histological subtypes (osteosarcoma, chondrosarcoma, Ewing sarcoma, and chordoma) in the propensity score-matched cohort. Significant survival differences were observed in osteosarcoma (*p* < 0.0001) and chondrosarcoma (*p* = 0.00014), but not in Ewing sarcoma (*p* = 0.38) or chordoma (*p* = 0.35).

**Figure 7:**
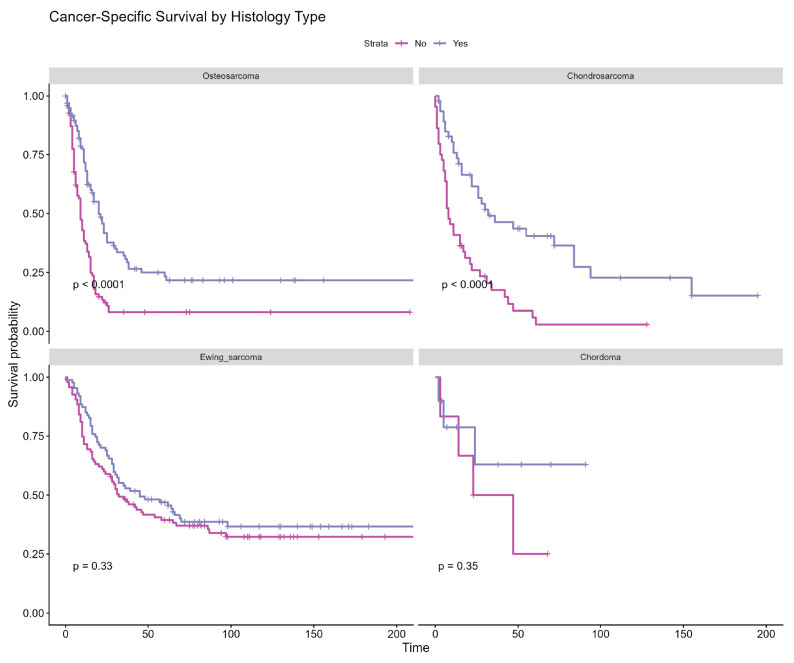
Kaplan–Meier curves for cancer-specific survival (CSS) stratified by primary tumor resection status across four histological subtypes (osteosarcoma, chondrosarcoma, Ewing sarcoma, and chordoma) in the propensity score-matched cohort. Significant survival differences were observed in osteosarcoma (*p* < 0.0001) and chondrosarcoma (*p* < 0.0001), but not in Ewing sarcoma (*p* = 0.33) or chordoma (*p* = 0.35).

**Figure 8:**
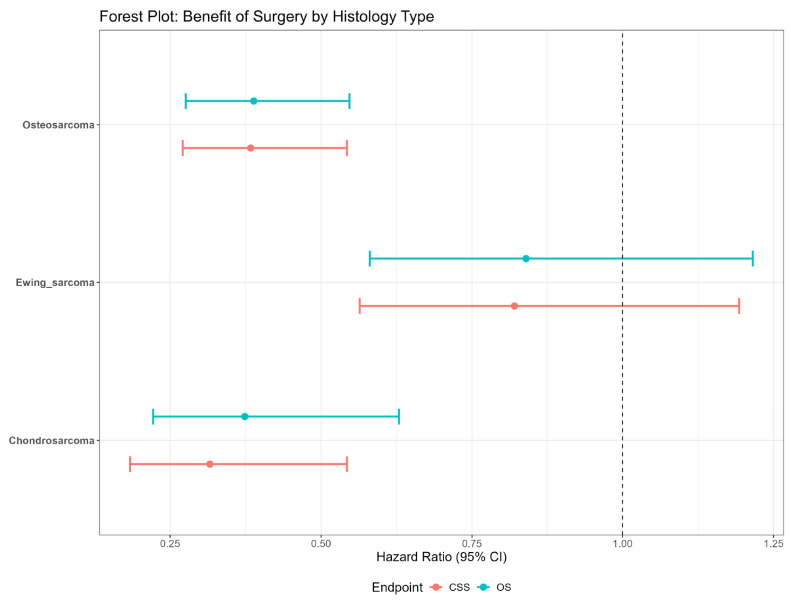
Forest plot of subgroup analysis illustrating the hazard ratios (HRs) and 95% confidence intervals (CIs) for the effect of primary tumor resection on overall survival (OS; teal) and cancer-specific survival (CSS; salmon) across histological subtypes in the propensity score-matched cohort. Three subtypes are shown (osteosarcoma, chondrosarcoma, and Ewing sarcoma); chordoma was excluded from multivariable analysis due to insufficient sample size. HRs were derived from multivariable Cox proportional hazards models adjusted for age, grade, T/N stage, and tumor size. The dashed vertical line indicates HR = 1.0 (no effect). The interaction *p*-value was calculated using the likelihood ratio test comparing models with and without a resection × histology interaction term.

Kaplan–Meier analysis within the PSM cohort revealed significant heterogeneity in the survival benefit of PTR across histological subtypes (Figure 6 and Figure 7). Osteosarcoma patients who underwent PTR demonstrated significantly improved OS and CSS compared with those who did not (both *p* < 0.0001). Similarly, chondrosarcoma patients showed significant survival benefit from PTR (OS: *p* = 0.00014; CSS: *p* < 0.0001). In contrast, no significant survival difference was observed between PTR and non-PTR groups in Ewing sarcoma or chordoma patients (all *p* > 0.05). Forest plot analysis of multivariable Cox models within each subtype confirmed these findings: HRs for osteosarcoma and chondrosarcoma were significantly less than 1.0 with 95% CIs entirely to the left of the null, while the HR for Ewing sarcoma favored surgery but did not reach statistical significance (Figure 8). Chordoma was excluded from multivariable subgroup analysis due to insufficient sample size. The likelihood ratio test for the PTR × histology interaction term was statistically significant, confirming that the surgical benefit is heterogeneous across subtypes.

It should be noted that the findings for chordoma and Ewing sarcoma should be interpreted with particular caution given the relatively limited sample sizes in these subgroups. The effect estimates for these subtypes may be unstable and more susceptible to random variation and residual confounding. These results are therefore considered exploratory and hypothesis-generating rather than definitive.

## 4. Discussion

The present study, leveraging a large population-based SEER cohort with PSM-controlled confounding, demonstrates that PTR is independently associated with a significant improvement in both OS and CSS in patients with synchronous metastatic PMBNs. In the matched cohort, PTR was associated with approximately 65–66% reduction in the risk of all-cause and cancer-specific mortality (OS HR = 0.34; CSS HR = 0.35), with high statistical significance. These findings were robust across both survival endpoints and persisted after multivariable adjustment for multiple clinical covariates. Importantly, the survival benefit of PTR was not uniform across histological subtypes: osteosarcoma and chondrosarcoma patients derived significant benefit, while Ewing sarcoma and chordoma patients did not show statistically significant improvement.

Our findings are broadly consistent with, and extend, prior SEER-based investigations of PTR in metastatic bone tumors. Malik et al. first demonstrated in a large SEER cohort that surgical resection of the primary site was associated with improved OS in metastatic PMBNs, though their analysis was limited by the absence of PSM and potential residual confounding [14]. Tong et al. subsequently applied PSM to a similar SEER cohort and confirmed the survival benefit of PTR, additionally developing a prediction model to identify patients most likely to benefit [16]. Hu et al. focused specifically on pelvic bone sarcomas with synchronous metastasis and similarly found that PTR improved survival [17]. Our study extends these findings by including a larger, more contemporary cohort (2004–2022), applying a more stringent PSM protocol, and conducting comprehensive histotype-stratified subgroup analyses with formal interaction testing. The survival benefit of PTR in the metastatic setting is not unique to bone tumors: analogous findings have been reported in metastatic soft-tissue sarcoma [23], metastatic lung cancer [24], de novo stage IV breast cancer [25], and metastatic differentiated thyroid cancer [26], suggesting a generalizable biological rationale for cytoreductive surgery across tumor types.

The heterogeneity of PTR benefit across histological subtypes is biologically plausible and clinically important. Osteosarcoma and chondrosarcoma are both characterized by relative resistance to systemic therapy, osteosarcoma responds to chemotherapy but metastatic disease remains difficult to control, while chondrosarcoma is largely chemoresistant and radioresistant, making surgery the primary modality for disease control even in advanced settings [12,27]. In this context, PTR may meaningfully reduce tumor burden and eliminate the primary source of metastatic seeding [28,29]. By contrast, Ewing sarcoma is highly chemosensitive and radiosensitive, and systemic therapy combined with local radiotherapy can achieve effective local control even without surgery in many cases [8]. The lack of significant PTR benefit in Ewing sarcoma may therefore reflect the adequacy of non-surgical local control in this subtype. For chordoma, the small sample size in our matched cohort (*n* = 16) precludes definitive conclusions, and the role of surgery in metastatic chordoma warrants dedicated investigation [30].

The role of primary tumor resection in patients with synchronous metastatic primary malignant bone neoplasms must be understood within the broader context of contemporary multidisciplinary sarcoma care. Surgical decision-making in this setting is highly individualized and should integrate histologic subtype, metastatic extent, systemic treatment options, local symptom control, patient performance status, and overall treatment goals. As highlighted by Samà et al. in their comprehensive review of mesenchymal tumor management, integrating surgical and non-surgical strategies across different clinical scenarios is essential for optimizing outcomes in advanced mesenchymal tumors [31]. The present findings reinforce the importance of individualized, multidisciplinary tumor board decision-making rather than a uniform surgical approach for all patients with metastatic primary malignant bone neoplasms.

Several biological mechanisms may underlie the survival benefit of PTR in PBMNs subtypes. First, cytoreduction through PTR directly reduces total tumor burden, potentially alleviating cancer-related systemic inflammation, immunosuppression, and cachexia [28,29]. Second, the primary tumor serves as a continuous source of circulating tumor cells that seed distant metastases; its removal may interrupt this process and reduce the rate of new metastatic lesion formation [28]. Third, primary tumors are known to induce systemic immunosuppression through the secretion of immunomodulatory cytokines and the recruitment of immunosuppressive cell populations; PTR may partially restore anti-tumor immune surveillance and enhance the efficacy of concurrent systemic therapies [29].

The strengths of this study include its large, population-based sample drawn from a high-quality national cancer registry; the application of PSM to reduce treatment selection bias; the use of dual survival endpoints (OS and CSS); the comprehensive histotype-stratified subgroup analysis with formal interaction testing; and the use of stratified Cox models to account for the matched-pair design. In addition, the inclusion of marital status as a covariate is supported by prior SEER-based studies demonstrating that marital status is an independent prognostic factor for survival in osteosarcoma, chondrosarcoma, and primary bone cancer [32,33,34,35]. These methodological features represent a meaningful advance over prior studies in this field.

Within the contemporary multidisciplinary management framework for advanced sarcomas, the decision to pursue primary tumor resection must be individualized based on histologic subtype, performance status, metastatic burden, systemic treatment options, and patient preferences. Samà et al. recently emphasized that integrating surgical and non-surgical strategies across different clinical scenarios is essential for optimizing outcomes in mesenchymal tumors, and that no single treatment modality should be applied uniformly across all patients [36]. Our findings are consistent with this framework: the significant survival benefit of PTR observed in osteosarcoma and chondrosarcoma, both relatively chemoresistant subtypes supports a role for cytoreductive surgery in carefully selected patients, while the lack of significant benefit in Ewing sarcoma reflects the adequacy of systemic therapy and radiotherapy for local control in this chemosensitive subtype.

Several important limitations must be acknowledged. First, as a retrospective observational study, residual confounding from unmeasured variables cannot be excluded. The SEER database lacks critical clinical information including patient performance status (e.g., ECOG score), metastatic burden and anatomical distribution, specific chemotherapy regimens and dose intensity, and details of metastasis-directed local therapy. Patients selected for PTR are likely to have had better performance status and lower metastatic burden than those managed non-operatively, factors that independently predict better survival and cannot be fully accounted for by PSM. Second, PSM can only balance measured confounders; unmeasured confounders remain a source of bias. Third, the small sample size of the chordoma subgroup limits the power of subgroup analyses for this histotype. Fourth, immortal time bias is an important potential concern in observational surgical studies [37,38]. In the SEER database, the exact timing of surgery relative to diagnosis is not precisely captured, and patients who underwent PTR must have survived long enough to receive the procedure. This introduces a period of “immortal time, during which the patient could not have died, that is attributed to the surgical group but not the non-surgical group, potentially inflating the apparent survival benefit of PTR [38,39]. Although PSM and multivariable adjustment partially mitigate this bias, it cannot be fully eliminated in the absence of time-to-surgery data. Future studies with detailed treatment timing information are needed to quantify and correct for this potential source of bias. Accordingly, substantial residual selection bias may remain despite PSM, and the observed association between primary tumor resection and improved survival should not be interpreted as definitive evidence of causality. More broadly, the histology-specific subgroup analyses should be interpreted cautiously, particularly for subgroups with relatively small sample sizes. The estimates for chordoma and Ewing sarcoma may be unstable and more susceptible to random variation and residual confounding. The results for these two subtypes should therefore be considered exploratory rather than confirmatory. The main clinical interpretation of this study should focus primarily on the larger and more representative histologic subgroups, osteosarcoma and chondrosarcoma, where sample sizes are sufficient to support more reliable effect estimates. Future dedicated studies with larger chordoma and Ewing sarcoma cohorts are needed to clarify the role of PTR in these subtypes.

Additionally, although PSM improved the balance of most baseline characteristics, some residual differences remained after matching, particularly in neoadjuvant and adjuvant radiotherapy use. These differences may reflect variations in tumor extent, resectability, local treatment strategy, or multidisciplinary decision-making, and may have influenced the observed association between primary tumor resection and survival outcomes. Specifically, differences in radiotherapy use after matching suggest that treatment pathways may still have differed between the PTR and non-PTR groups. Radiotherapy, particularly in Ewing sarcoma and chordoma, can serve as an effective alternative to surgery for local control, and its unequal distribution between groups may have partially confounded the estimated survival benefit of PTR. This residual imbalance should be considered when interpreting the reported survival benefit associated with primary tumor resection.

The clinical implications of this study are nuanced. Our results do not support routine PTR for all patients with synchronous metastatic PMBNs. Rather, they suggest that for carefully selected patients, particularly those with osteosarcoma or chondrosarcoma, adequate performance status, and manageable metastatic burden, PTR should be considered as a component of a comprehensive, multidisciplinary treatment strategy. Future prospective multicenter cohort studies with detailed clinical data collection are needed to validate these findings and to identify biomarkers that predict surgical benefit. Given the ethical and practical challenges of conducting randomized controlled trials in this rare disease population, high-quality real-world evidence from well-designed observational studies remains an essential source of clinical guidance.

## 5. Conclusions

In this large population-based, PSM-controlled analysis, PTR was independently associated with significantly improved OS and CSS in patients with synchronous metastatic PMBNs, with the greatest benefit observed in osteosarcoma and chondrosarcoma. These findings provide important evidence to support individualized, multidisciplinary surgical decision-making in this challenging patient population, while underscoring the need for prospective validation.

## Figures and Tables

**Table 1:** Baseline demographic and clinical characteristics of patients with synchronous metastatic primary malignant bone neoplasms before propensity score matching (PSM).

Characteristic	Overall *n* = 1046	No Resection *n* = 388	Resection *n* = 658	*p*-Value
Age group				<0.001
<20 years	494 (47.23%)	127 (32.73%)	367 (55.78%)	
20–59 years	389 (37.19%)	185 (47.68%)	204 (31.00%)	
≥60 years	163 (15.58%)	76 (19.59%)	87 (13.22%)	
Sex				0.624
Female	389 (37.19%)	148 (38.14%)	241 (36.63%)	
Male	657 (62.81%)	240 (61.86%)	417 (63.37%)	
Race				0.388
White	847 (80.98%)	309 (79.64%)	538 (81.76%)	
Black	99 (9.46%)	43 (11.08%)	56 (8.51%)	
Others	100 (9.56%)	36 (9.28%)	64 (9.73%)	
Marital status				0.954
Married	282 (26.96%)	105 (27.06%)	177 (26.90%)	
Others	764 (73.04%)	283 (72.94%)	481 (73.10%)	
Histological type				<0.001
Osteosarcoma	542 (51.82%)	143 (36.86%)	399 (60.64%)	
Chondrosarcoma	188 (17.97%)	70 (18.04%)	118 (17.93%)	
Ewing sarcoma	295 (28.20%)	166 (42.78%)	129 (19.60%)	
Chordoma	21 (2.01%)	9 (2.32%)	12 (1.82%)	
Primary site				<0.001
Axial	395 (37.76%)	234 (60.31%)	161 (24.47%)	
Extremity	651 (62.24%)	154 (39.69%)	497 (75.53%)	
Grade				<0.001
I–II	62 (5.93%)	19 (4.90%)	43 (6.53%)	
III–IV	509 (48.66%)	125 (32.22%)	384 (58.36%)	
Unknown	475 (45.41%)	244 (62.89%)	231 (35.11%)	
T stage				0.136
T1	231 (22.08%)	97 (25.00%)	134 (20.36%)	
T2	717 (68.55%)	249 (64.18%)	468 (71.12%)	
T3	83 (7.93%)	36 (9.28%)	47 (7.14%)	
T4	15 (1.43%)	6 (1.55%)	9 (1.37%)	
N stage				<0.001
N0	912 (87.19%)	319 (82.22%)	593 (90.12%)	
N1	134 (12.81%)	69 (17.78%)	65 (9.88%)	
Tumor size				0.096
<5 cm	75 (7.17%)	33 (8.51%)	42 (6.38%)	
5–15 cm	738 (70.55%)	281 (72.42%)	457 (69.45%)	
>15 cm	233 (22.28%)	74 (19.07%)	159 (24.16%)	
Chemotherapy				0.265
No/Unknown	161 (15.39%)	66 (17.01%)	95 (14.44%)	
Yes	885 (84.61%)	322 (82.99%)	563 (85.56%)	
Radiotherapy				<0.001
No/Unknown	695 (66.44%)	185 (47.68%)	510 (77.51%)	
Yes	351 (33.56%)	203 (52.32%)	148 (22.49%)	
Neoadjuvant radiotherapy				0.003
No/Unknown	1033 (98.76%)	388 (100.00%)	645 (98.02%)	
Yes	13 (1.24%)	0 (0.00%)	13 (1.98%)	
Adjuvant radiotherapy				<0.001
No/Unknown	894 (85.47%)	372 (95.88%)	522 (79.33%)	
Yes	152 (14.53%)	16 (4.12%)	136 (20.67%)	

Data are presented as *n* (%). *p*-values from Pearson’s chi-squared test or Fisher’s exact test.

**Table 2:** Baseline demographic and clinical characteristics of patients with synchronous metastatic primary malignant bone neoplasms after propensity score matching (PSM).

Characteristic	Overall *n* = 488	No Resection *n* = 244	Resection *n* = 244	*p*-Value
Age group				0.812
<20 years	199 (40.78%)	103 (42.21%)	96 (39.34%)	
20–59 years	209 (42.83%)	102 (41.80%)	107 (43.85%)	
≥60 years	80 (16.39%)	39 (15.98%)	41 (16.80%)	
Sex				0.705
Female	174 (35.66%)	89 (36.48%)	85 (34.84%)	
Male	314 (64.34%)	155 (63.52%)	159 (65.16%)	
Race				0.911
White	383 (78.48%)	193 (79.10%)	190 (77.87%)	
Black	50 (10.25%)	25 (10.25%)	25 (10.25%)	
Others	55 (11.27%)	26 (10.66%)	29 (11.89%)	
Marital status				>0.999
Married	136 (27.87%)	68 (27.87%)	68 (27.87%)	
Others	352 (72.13%)	176 (72.13%)	176 (72.13%)	
Histological type				0.693
Osteosarcoma	199 (40.78%)	99 (40.57%)	100 (40.98%)	
Chondrosarcoma	91 (18.65%)	44 (18.03%)	47 (19.26%)	
Ewing sarcoma	182 (37.30%)	95 (38.93%)	87 (35.66%)	
Chordoma	16 (3.28%)	6 (2.46%)	10 (4.10%)	
Primary site				0.856
Axial	238 (48.77%)	120 (49.18%)	118 (48.36%)	
Extremity	250 (51.23%)	124 (50.82%)	126 (51.64%)	
Grade				0.256
I–II	40 (8.20%)	15 (6.15%)	25 (10.25%)	
III–IV	202 (41.39%)	103 (42.21%)	99 (40.57%)	
Unknown	246 (50.41%)	126 (51.64%)	120 (49.18%)	
T stage				0.950
T1	123 (25.20%)	62 (25.41%)	61 (25.00%)	
T2	317 (64.96%)	160 (65.57%)	157 (64.34%)	
T3	42 (8.61%)	19 (7.79%)	23 (9.43%)	
T4	6 (1.23%)	3 (1.23%)	3 (1.23%)	
N stage				0.313
N0	414 (84.84%)	211 (86.48%)	203 (83.20%)	
N1	74 (15.16%)	33 (13.52%)	41 (16.80%)	
Tumor size				0.873
<5 cm	46 (9.43%)	24 (9.84%)	22 (9.02%)	
5–15 cm	350 (71.72%)	176 (72.13%)	174 (71.31%)	
>15 cm	92 (18.85%)	44 (18.03%)	48 (19.67%)	
Chemotherapy				0.259
No/Unknown	75 (15.37%)	33 (13.52%)	42 (17.21%)	
Yes	413 (84.63%)	211 (86.48%)	202 (82.79%)	
Radiotherapy				0.714
No/Unknown	280 (57.38%)	138 (56.56%)	142 (58.20%)	
Yes	208 (42.62%)	106 (43.44%)	102 (41.80%)	
Neoadjuvant radiotherapy				0.004 *
No/Unknown	479 (98.16%)	244 (100.00%)	235 (96.31%)	
Yes	9 (1.84%)	0 (0.00%)	9 (3.69%)	
Adjuvant radiotherapy				<0.001 *
No/Unknown	387 (79.30%)	237 (97.13%)	150 (61.48%)	
Yes	101 (20.70%)	7 (2.87%)	94 (38.52%)	

Data are presented as *n* (%). *p*-values from Pearson’s chi-squared test or Fisher’s exact test. * Remained statistically significant after PSM, indicating residual imbalance.

**Table 3:** Univariable and multivariable Cox proportional hazards regression analyses for overall survival (OS) in patients with synchronous metastatic primary malignant bone neoplasms after propensity score matching (PSM).

Variable	Category	*n* (%)	HR (Univariable)	HR (Multivariable)
Resection	No	244 (50.0%)	Reference	Reference
	Yes	244 (50.0%)	0.62 (0.50–0.77, ***p* < 0.001**)	0.34 (0.21–0.54, ***p* < 0.001**)
Age group	<20	199 (40.8%)	Reference	Reference
	20–59	209 (42.8%)	1.71 (1.35–2.16, ***p* < 0.001**)	1.46 (0.49–4.41, *p* = 0.497)
	≥60	80 (16.4%)	2.02 (1.50–2.72, ***p* < 0.001**)	3.04 (0.65–14.17, *p* = 0.156)
Marital status	Married	136 (27.9%)	Reference	Reference
	Others	352 (72.1%)	0.60 (0.48–0.76, ***p* < 0.001**)	0.79 (0.35–1.77, *p* = 0.562)
Histological type	Osteosarcoma	199 (40.8%)	Reference	Reference
	Chondrosarcoma	91 (18.6%)	0.82 (0.62–1.09, *p* = 0.173)	0.82 (0.39–1.72, *p* = 0.597)
	Ewing sarcoma	182 (37.3%)	0.43 (0.33–0.55, ***p* < 0.001**)	0.68 (0.35–1.34, *p* = 0.267)
	Chordoma	16 (3.3%)	0.39 (0.18–0.83, ***p* = 0.015**)	0.13 (0.02–0.93, ***p* = 0.042**)
Grade	I–II	40 (8.2%)	Reference	Reference
	III–IV	202 (41.4%)	1.42 (0.96–2.10, *p* = 0.078)	2.61 (1.00–6.77, ***p* = 0.049**)
	Unknown	246 (50.4%)	1.01 (0.69–1.50, *p* = 0.945)	2.35 (0.81–6.79, *p* = 0.115)
T stage	T1	123 (25.2%)	Reference	Reference
	T2	317 (65.0%)	1.12 (0.87–1.44, *p* = 0.366)	1.49 (0.83–2.66, *p* = 0.178)
	T3	42 (8.6%)	1.46 (0.95–2.23, *p* = 0.087)	1.87 (0.64–5.40, *p* = 0.250)
	T4	6 (1.2%)	1.97 (0.79–4.86, *p* = 0.144)	0.19 (0.02–2.00, *p* = 0.168)
Radiotherapy	No/Unknown	280 (57.4%)	Reference	Reference
	Yes	208 (42.6%)	0.66 (0.53–0.82, ***p* < 0.001**)	0.90 (0.38–2.11, *p* = 0.805)
Adjuvant radiotherapy	No/Unknown	387 (79.3%)	Reference	Reference
	Yes	101 (20.7%)	0.65 (0.50–0.85, ***p* = 0.001**)	1.56 (0.75–3.25, *p* = 0.231)

*n* = 488, events = 351; Likelihood ratio test = 56.26 on 14 df (*p* < 0.001); Model stratified by matched pairs (subclass). Bold values indicate statistical significance (*p* < 0.05).

**Table 4:** Univariable and multivariable Cox proportional hazards regression analyses for cancer-specific survival (CSS) in patients with synchronous metastatic primary malignant bone neoplasms after propensity score matching (PSM).

Variable	Category	*n* (%)	HR (Univariable)	HR (Multivariable)
Resection	No	244 (50.0%)	Reference	Reference
	Yes	244 (50.0%)	0.59 (0.48–0.74, ***p* < 0.001**)	0.35 (0.22–0.55, ***p* < 0.001**)
Age group	<20	199 (40.8%)	Reference	Reference
	20–59	209 (42.8%)	1.69 (1.33–2.14, ***p* < 0.001**)	1.82 (0.61–5.49, *p* = 0.284)
	≥60	80 (16.4%)	1.91 (1.41–2.60, ***p* < 0.001**)	3.34 (0.72–15.40, *p* = 0.122)
Marital status	Married	136 (27.9%)	Reference	Reference
	Others	352 (72.1%)	0.60 (0.48–0.76, ***p* < 0.001**)	0.94 (0.43–2.07, *p* = 0.887)
Histological type	Osteosarcoma	199 (40.8%)	Reference	Reference
	Chondrosarcoma	91 (18.6%)	0.80 (0.60–1.06, *p* = 0.123)	0.83 (0.39–1.76, *p* = 0.632)
	Ewing sarcoma	182 (37.3%)	0.43 (0.34–0.56, ***p* < 0.001**)	0.74 (0.38–1.45, *p* = 0.379)
	Chordoma	16 (3.3%)	0.40 (0.19–0.86, ***p* = 0.019**)	0.11 (0.01–0.94, ***p* = 0.043**)
Grade	I–II	40 (8.2%)	Reference	Reference
	III–IV	202 (41.4%)	1.48 (0.99–2.22, *p* = 0.057)	2.52 (0.97–6.60, *p* = 0.059)
	Unknown	246 (50.4%)	1.04 (0.69–1.55, *p* = 0.864)	2.45 (0.86–6.96, *p* = 0.093)
Chemotherapy	No/Unknown	75 (15.4%)	Reference	Reference
	Yes	413 (84.6%)	0.76 (0.57–1.03, *p* = 0.075)	0.80 (0.37–1.73, *p* = 0.568)
Radiotherapy	No/Unknown	280 (57.4%)	Reference	Reference
	Yes	208 (42.6%)	0.67 (0.54–0.84, ***p* < 0.001**)	0.97 (0.41–2.25, *p* = 0.938)
Adjuvant radiotherapy	No/Unknown	387 (79.3%)	Reference	Reference
	Yes	101 (20.7%)	0.65 (0.49–0.85, ***p* = 0.002**)	1.44 (0.70–2.99, *p* = 0.325)

*n* = 488, events = 338; Likelihood ratio test = 50.21 on 12 df (*p* < 0.001); Model stratified by matched pairs (subclass). Bold values indicate statistical significance (*p* < 0.05).

## Data Availability

The original contributions presented in this study are included in the article. Further inquiries can be directed to the corresponding authors.

## Supplementary Materials

The following supporting information can be downloaded at: https://www.mdpi.com/article/10.3390/cancers18142201/s1, Figure S1: Covariate balance plot before and after propensity score matching (PSM). Standardized mean differences (SMDs) for all covariates included in the propensity score model are shown before PSM (open circles) and after PSM (filled circles). An SMD < 0.1 indicates adequate balance. After matching, all covariates achieved SMD < 0.1, indicating satisfactory covariate balance between the resection and non-resection groups.
