# Supplementary material for: Primary Tumor Resection and Survival Benefit in Patients with Synchronous Metastatic Primary Malignant Bone Neoplasms: A Propensity Score-Matched Analysis of the SEER Database
Source: Cancers (Basel). 2026 Jul 8;18(14):2201. doi: 10.3390/cancers18142201 (PMC13406352; doi:10.3390/cancers18142201)
Supplement: Supplementary file 1 [file cancers-18-02201-s001.zip › cancers-4370574-supplementary.pdf]

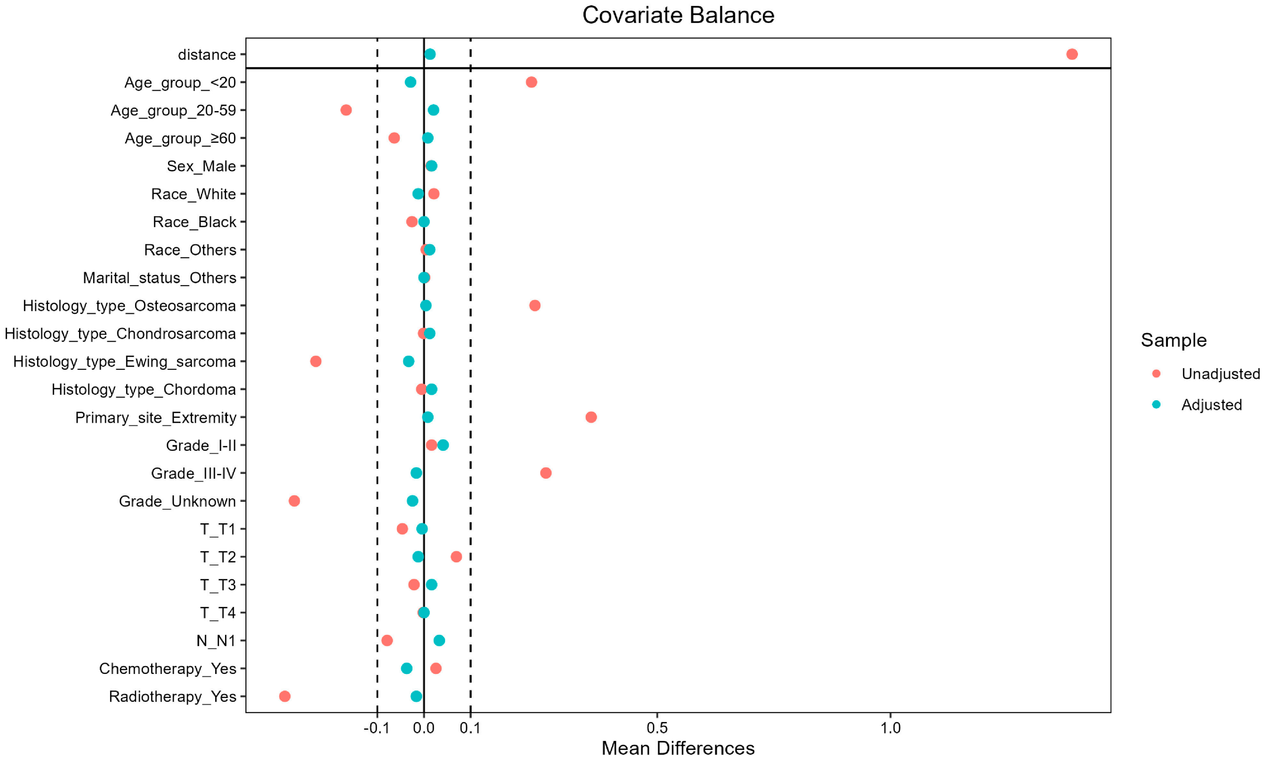

Figure S1. Covariate balance plot before and after propensity score matching (PSM). Standardized mean differences (SMDs) for all covariates included in the propensity score model are shown before PSM (open circles) and after PSM (filled circles). An SMD < 0.1 indicates adequate balance. After matching, all covariates achieved SMD < 0.1, indicating satisfactory covariate balance between the resection and non-resection groups.
